# Total anomalous pulmonary venous connection in 80 patients: Primary sutureless repair and outcomes

**DOI:** 10.3389/fsurg.2022.1086596

**Published:** 2023-01-11

**Authors:** Gefei Li, Baoying Meng, Cheng Zhang, Weimin Zhang, Xiaodong Zhou, Qing Zhang, Yiqun Ding

**Affiliations:** ^1^ Department of Pediatric Cardiothoracic Surgery, Shenzhen Children's Hospital, Shenzhen, China; ^2^ Department of Pediatric Cardiology, The University of Hong Kong-Shenzhen Hospital, Shenzhen, China

**Keywords:** total anomalous pulmonary venous connection, TAPVC, sutureless repair, congenital heart disease, pulmonary venous obstruction

## Abstract

**Introduction:**

Total anomalous pulmonary venous connection (TAPVC) is a rare but critical cardiac anomaly, in which pulmonary veins are connected to an abnormal location rather than the left atrium. The prognosis can be extremely poor without intervention, with a mortality of 80% during infancy. The purpose of this research is to summarize the outcomes and relevant risk factors of 80 total anomalous pulmonary venous connection (TAPVC) patients who underwent primary TAPVC sutureless repair and discuss the indications and benefits of primary sutureless repair.

**Methods:**

This retrospective review included 80 patients with TAPVC who underwent primary sutureless repair at a single institution between January 2015 and December 2020. Patients were subdivided into 4 groups according to Darling's classification. Risk factors that increase the postoperative pulmonary vein flow velocity were explored by Multiple Linear regression.

**Results:**

Anatomic TAPVC subtypes included supracardiac 35 (43.8%), cardiac 24 (30%), infracardiac 17 (21.2%), and mixed 4 (5%). Median age at repair was 16.5 days and median weight was 3.5 kg. Preoperative pulmonary venous obstruction (PVO)was presented in 20 (25%) patients. There were 2 early deaths and 1 late death. 2 patients developed postoperative PVO and none required reintervention. Prolonged cardiopulmonary bypass time (CPB) (*p* = 0.009), preoperative pneumonia (*p* = 0.022) and gender (*p* = 0.041) were found to be associated with the increase of postoperative pulmonary vein flow velocity.

**Discussion:**

Under the primary sutureless technique, no statistical difference was observed among the 4 subgroups in terms of postoperative pulmonary vein flow velocity (*p* = 0.589). The primary sutureless technique may eliminate the differences between subtypes while decrease the postoperative PVO rate, which makes it applicable in any subtypes of TAPVC. Following the favorable outcomes in preventing postoperative PVO in all subtypes in this study, we advocate the indications for primary sutureless repair may expand further to all the TAPVC patients.

## Introduction

Total anomalous pulmonary venous connection (TAPVC) is a rare but critical cardiac anomaly, in which pulmonary veins are connected to an abnormal location rather than the left atrium. Accounting for 1% to 3% of the congenital heart disease cases (1). The prognosis can be extremely poor without intervention, with a mortality of 80% during infancy (1, 2). The only treatment is surgical correction at an early stage. With developments in cardiac surgery, the surgical mortality is now generally below 10% (3, 4). Criteria that evaluate surgical outcomes of TAPVC usually include operative mortality rates and incidence of recurrent pulmonary venous obstruction. In previous studies, variables such as other coexisting cardiac anomalies, younger age at the time of repair, infracardiac and mixed TAPVC, and preoperative PVO were associated with a poorer prognosis (5–8). In the 1990s, sutureless pericardial repair was proposed to relieve PVO after TAPVC repair (9–11). Though there is a potential risk of bleeding from the gap between the confluence and pericardium into the posterior mediastinum or pleural cavity, this technique was associated with a favorable survival rate for reintervention of postoperative PVO (11, 12). Thus, the indications of sutureless repair for TAPVC as a primary operation have been gradually expanded throughout the years (13–16). In this study, we have retrospectively collected data from a contemporary cohort of patients with TAPVC who all underwent primary sutureless repair to evaluate the surgical outcomes and identify variables that increase postoperative pulmonary vein flow velocity.

## Materials and methods

### Ethics statement

The Institutional Review Board of Shenzhen Children's hospital approved this study and the need for individual consent was waived due to the retrospective nature of the study.

### Study population and definitions

Patients with TAPVC who underwent primary sutureless repair between January 2015 and December 2020 at Shenzhen Children's Hospital were consecutively enrolled. All the TAPVC patients that admitted in our center underwent primary sutureless repair during this study period. A review of medical records, including medical history, clinical examination records, operative details, and ICU charts were performed. Patients with single ventricle or heterotaxy were excluded. The classification of TAPVC was based on that proposed by Darling in 1957 as supracardiac, cardiac, infracardiac, and mixed types (17). The diagnosis was mainly made by echocardiography, 66 cases also performed computed tomography (CT) for detailed morphology. Primary sutureless repair was required for all patients present with or without preoperative PVO. Emergency surgery referred to the operation performed within the first 24 h after presentation to save life. Early death was defined as death within 30 days of operation or during primary hospitalization. Other deaths were defined as late deaths. Reintervention was defined as any operation performed secondary to recurrent PVO. Pulmonary veins were deemed obstructed by a nonphasic flow rate of >1.8 m/s based on echocardiography (8).

### Operative data

Primary sutureless repair was performed through a median sternotomy under standard cardiopulmonary bypass (CPB) in 80 patients. Among them, deep hypothermic circulatory arrest (DHCA) was used in 55 patients.We adopt DHCA for most of the cases because the heart needs to be rotated in our sutureless technique, and DHCA allows us to obtain a satisfactory surgical exposure and avoid distortion of the cannula. The ductus arteriosus was dissected and ligated before CPB was started if existed. All operations were performed by the same group of surgeons led by the same head surgeon. Surgeons'multilevel effects didn't exist in this study. A part of the surgeons moved to another hospital after we finished the study cohort. All patient data come from Shenzhen Children's hospital.

### Sutureless technique

For supracardiac and infracardiac TAPVC, the heart would be positioned under the right hemi-sternum or in the right thoracic cavity after being rotated toward the patient's right. An incision on the common pulmonary vein was made and extended to each individual pulmonary vein, also to the vertical vein beyond every stenotic segment, the incision was continued toward the pleural pericardial reflection laterally. An incision was made on the posterior wall of the left atrium. The left atrial wall was anastomosed with the pericardium adjacent to the pulmonary vein, avoiding direct contact with the venous wall. The atrial septal defect (ASD) was closed with/without a pericardial patch. The vertical vein was ligated prior to sternal closure if the intra-operative echocardiogram result is satisfied, and the hemodynamics were stable. For mixed TAPVC, we combined the sutureless technique, other anastomosis techniques and intra atrium baffle technique to connect all pulmonary veins to the left atrium, avoiding or reducing trauma to the pulmonary venous endothelium. In this study, there were 4 mixed type TAPVC patients. One of them had 3 supracardiac pulmonary veins combined with 1 infracardiac pulmonary vein. Other 3 mixed type patients all had three intracardiac pulmonary veins combined with one supracardiac pulmonary vein. In the case that combines supracardiac and infracardiac type, we also cover the atrium on the incisions of the four pulmonary veins with no big differences than what we described above. For the cases that own three intracardiac pulmonary veins combining with one supracardiac pulmonary vein, We would do a parallel anastomosis of the supracardiac pulmonary vein with the auricula. The stitches would be on the pericardium tissues around the pulmonary vein, and the pulmonary venous endothelium would also be intact. For cardiac TAPVC we also adopt our sutureless technique which doesn't contact the endothelium. For the cardiac type cases with single or multiple stenosis before flow into coronary sinus, we use similar sutureless technique as the supracardiac ones.

### Statistical analysis

Statistical analyses were performed with SPSS24. Continuous variables were reported as mean ± standard deviation or medians with range or interquartile range (IQR) and categorical variables as absolute numbers or percentages. Due to the very low incidence of adverse outcomes,we did not choose Cox regression or Logistic regression for analysis. Instead, we first apply simple linear regression for all the factors that we record. Then we chose the factors with *p* < 0.2 into multiple linear regression model for further analysis. The final risk factors that increase the postoperative pulmonary vein flow velocity were determined by the Multiple linear regression model. Take *α* = 0.05 as examination standard.

## Results

### Baseline characteristics

Between 2015 and 2021, A total of 80 TAPVC patients underwent primary sutureless TAPVC repair. Of the 80 patients, 47 were male (58.8%) and 33 were female (41.2%). Median age at time of surgery was 16.5 days (range 0 to 270 days) with median weight 3.5 kg (range 2.2 to 7.6 kg). Anatomic subtypes presented as, supracardiac 35 (43.8%), cardiac 24 (30%), infracardiac 17 (21.2%), and mixed 4 (5%). Preoperative PVO was found in 20 patients (25%), including 9 in supracardiac, 3 in cardiac and 8 in infracardiac subtypes. Detailed patient baseline characteristics are illustrated in Table 1.

**Table 1:** Patient characteristics.

Covariate	Total = 80
**Age at surgery (days)**	
Mean(SD)	47.8 (64.6)
Median (IQR)	16.5 (7–62.75)
Weight at surgery (kg)	
Mean (SD)	3.9 (1.3)
Median (IQR)	3.5 (3–4.37)
Gender, *n* (%)	
Male	47 (58.8)
Female	33 (41.2)
TAPVC type, *n* (%)	
Supracardiac	35 (43.8)
Cardiac	24 (30.0)
Infracardiac	17 (21.2)
Mixed	4 (5.0)
Preoperative PVO, *n* (%)	
Supracardiac	9 (25.7)
Cardiac	3 (12.5)
Infracardiac	8 (47.1)
Mixed	0 (0)

### In-hospital results

There were 20 (25.0%) cases combined with preoperative pneumonia. 19 (23.8%) cases had preoperative ventilator support. 8(10.0%) cases underwent emergency surgery. The mean CPB and aortic cross-clamping times (ACC) for the entire cohort were 88.5 ± 35.7 and 36.2 ± 13.2 min. DHCA was used in 55(68.8%) patients with a median ischemic time of 31 min (range: 10–65). The median duration of stay at the cardiac intensive care unit (CICU) and hospital were 4 days (IQR, 3–6) and 12 days (IQR, 10–15). Detailed In-hospital information is shown in Table 2.

**Table 2:** In-hospital and follow-up variables.

Covariate	Total = 80
Preoperative pneumonia, *n* (%)	20 (25.0%)
Preoperative ventilator support, *n* (%)	19 (23.8%)
Emergency surgery	8 (10.0%)
**CPB (minutes)**	
Mean ± SD	88.5 ± 35.7
Median	87.0
Minimum, maximum	42,333
**ACC (minutes)**	
Mean ± SD	36.2 ± 13.2
Median	32.5
Minimum, maximum	19,108
**DHCA, *n* (%)**	
No	25 (31.2)
Yes	55 (68.8)
**DHAC (minutes)**	
Mean ± SD	33.4 ± 1.3
Median	31
Minimum, maximum	10,65
**Mortality, *n* (%)**	
No	77 (96.2%)
Yes	3 (3.8%)
**Postoperative PVO, *n* (%)**	
No	78 (97.5%)
Yes	2 (2.5%)
**CICU stay (days)**	
Median (IQR)	4 (3–6)
Minimum, maximum	2,29
**Hospital stay (days)**	
Median (IQR)	12 (10–15)
Minimum, maximum	8,56

### Follow-up

**Figure 1:**
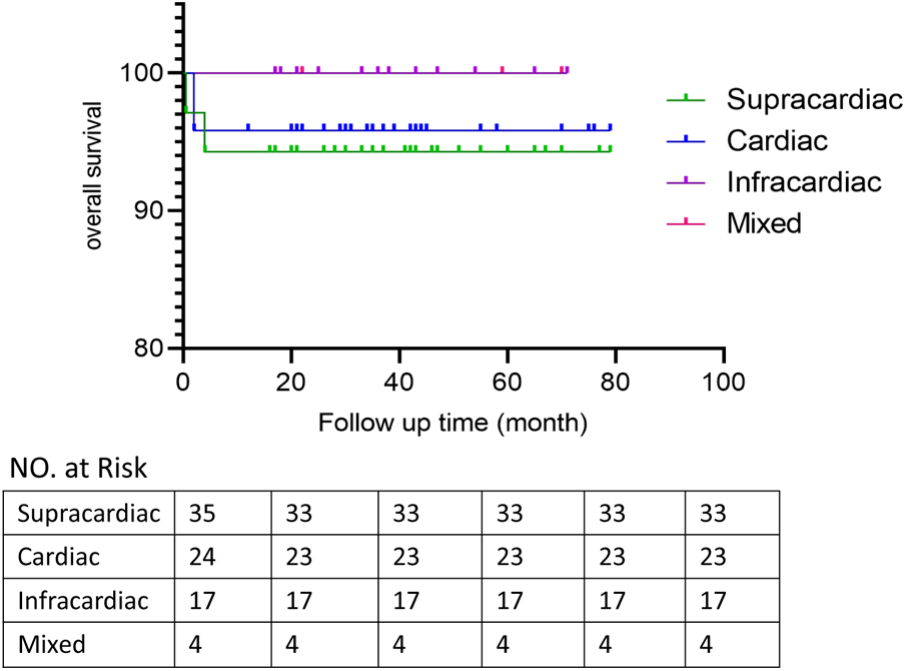
Overall survival time.

**Figure 2:**
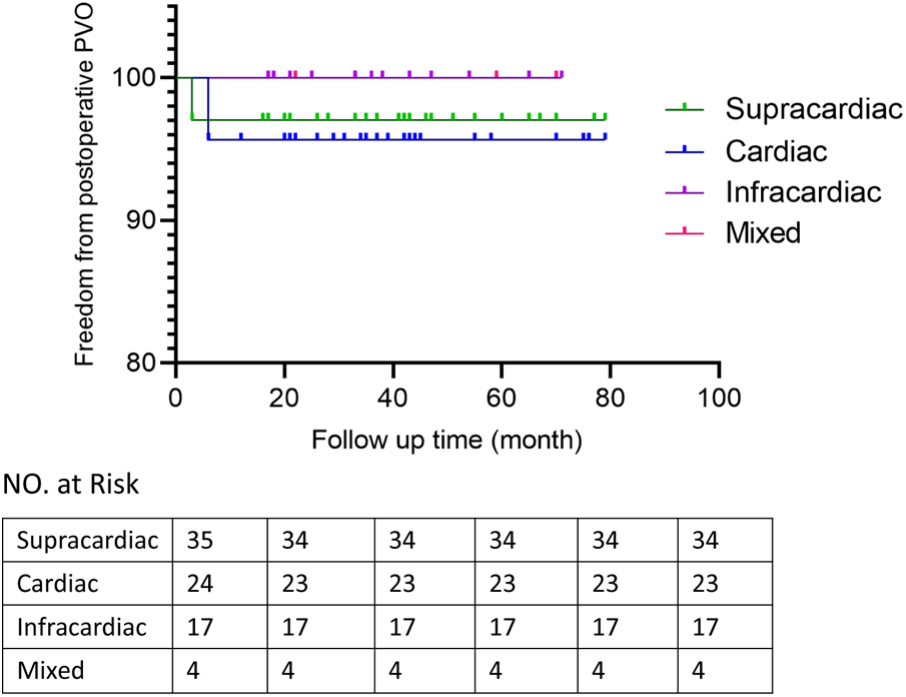
Freedom from postoperative PVO.

Patients discharged were followed up at 1, 3 and 6 months after surgery and then annually mainly by echocardiography to evaluate the postoperative pulmonary vein flow acceleration. We use echocardiography instead of CT scans because it provides not only steady images of the vessels, but also color doppler that enables us to measure the flow velocity of certain vessels. It is more accurate in detecting accelerated vein flow velocity. CT scan also has certain radiation, thus we want to avoid it during regular follow-up.At time of presentation, 2 patients (2.5%) had evidence of obstruction. 1 from spracardiac type and 1 from cardiac type. For patient 1, The latest echo report shows the right pulmonary vein (PV) flow velocity was 2.6 m/s, the velocity of left PV was 1.8 m/s. For patient 2, The latest echo report shows the left PV flow velocity was 2.1 m/s, the velocity of right PV was 1.7 m/s. Reintervention criteria in our center are based on the clinical manifestations of the patients. If symptoms like dyspenea, three concave signs, need for ventilation, failure to thrive, and limitation of activity appears, a reintervention surgery would be done. Both patients are under close observation, there is no further acceleration of their PV flow velocity throughout our follow-ups. And both were asymptomatic with normal growth and development, thus none needed reintervention currently. They are still under close observation and regular follow-up to monitor any change in PV velocity and symptoms. If they develop any of the above symptoms, a reintervention would be done. 2 patients presented early deaths, with the PV velocity of 1.3 m/s and 1.5 m/s respectively according to the death case discussion records. One due to aspiration led by improper feeding by the patient's mother. One due to acute pulmonary edema which led by pulmonary hypertension crisis. 1 late death occurred after the patient's second surgery because of the comprehensive effect of multiple conditions of the patient: Aortic dysplasia, severe pulmonary hypertension, severe bilateral pulmonary hypoplasia, tracheomalacia, and severe malnutrition. The patient never have a PVO, according to the the most recent echo report prior to death, the PV velocity was1.1 m/s.The overall outcomes of this cohort of patients are shown in Figures 1, 2. Detailed follow-up information is shown in Table 2.

### Risk factors that increase the postoperative pulmonary vein flow velocity

Prolonged CPB time (*p* = 0.009), preoperative pneumonia (*p* = 0.022) and gender (*p* = 0.041) were found to be associated with the increase of postoperative pulmonary vein flow velocity. Of note, under the primary sutureless technique, no statistical difference was observed among the 4 types in terms of postoperative pulmonary vein flow velocity (*p* = 0.589) and no statistical difference was observed in preoperative PVO group (*p* = 0.114). Detailed analysis results are showed in Tables 3, 4.

**Table 3:** Simple linear regression analysis of factors associated With postoperative pulmonary vein flow acceleration.

Variable	Estimate	Std Error	*t*	*P* Value	95%CI
TAPVC types	−0.019	0.036	−0.542	0.589	−0.091–0.052
Preoperative PVO	0.207	0.071	2.893	0.005	0.064–0.349
Age	0.0	0.001	−0.907	0.367	−0.001–0.001
Weight	−0.037	0.025	−1.466	0.147	−0.087–0.013
Gender	−0.128	0.065	−1.965	0.053	−0.257–0.002
Preoperative pneumonia	0.120	0.074	1.619	0.110	−0.028–0.267
Preoperative ventilation	0.098	0.077	1.268	0.209	−0.056–0.252
Emergency surgery	0.093	0.108	0.858	0.394	−0.122–0.308
DHCA	0.109	0.069	1.573	0.120	−0.029–0.247
CPB time	0.003	0.001	3.707	<0.001	0.001–0.005
ACC time	0.007	0.002	2.839	0.006	0.002–0.011
CICU stay	0.019	0.007	2.773	0.007	0.005–0.032
Hospital stay	0.013	0.005	2.756	0.007	0.004–0.022

**Table 4:** Multiple linear regression model of factors associated With postoperative pulmonary vein flow acceleration.

Variable	Estimate	Std Error	*t*	*P* Value	95%CI
Preoperative PVO	0.114	0.071	1.600	0.114	−0.028–0.255
Weight	−0.031	0.025	−1.258	0.213	−0.080–0.018
Gender	−0.125	0.060	−2.079	0.041	−0.244– (−0.005)
Preoperative pneumonia	0.166	0.071	2.342	0.022	0.025–0.307
DHCA	0.002	0.068	0.025	0.980	−0.135–0.138
CPB time	0.004	0.002	2.708	0.009	0.001–0.008
ACC time	−0.006	0.004	−1.389	0.169	−0.015–0.003
CICU stay	−0.001	0.011	−0.058	0.954	−0.023–0.022
Hospital stay	0.008	0.007	1.132	0.261	−0.006–0.023

## Discussion

From this TAPVC patient cohort from Shenzhen Children's Hospital, we have the following main findings: (1) the most common type was supracardiac, followed by cardiac and infracardiac; (2) Under the primary sutureless repair condition, subtypes like infracardiac, mixed and preoperative PVO might no longer be associated with the postoperative pulmonary vein flow velocity. (3) Prolonged CPB time (*p* = 0.009) and preoperative pneumonia (*p* = 0.022) were mainly associated with the increase of postoperative pulmonary vein flow velocity.

Supracardiac TAPVC is the most common type reported, accounting for about 40% to 45% of the total (3, 18, 19). Our study was consisted of supracardiac 35 (43.8%), cardiac 24 (30%), which echoed these observations, with cardiac type as the second most common type. On the contrary, some other series identified infracardiac type as the second most common type, accounting for about 20% to 26%.

Primary sutureless repair of biventricular TAPVC was associated with decreased mortality and postoperative PVO rate (20, 21). Sutureless technique anastomosing the left atrium to the posterior pericardium rather than to the pulmonary vein tissue itself. The key points for satisfactory outcomes of this technique include: fully relieving the preoperative pulmonary venous obstruction, atriopericardial anastomosis and avoidance of trauma to the pulmonary veins (22). It is a key technique to overcome postoperative PVO and lower the mortality rate. The diagnosis of PVO is usually made by echocardiography, with fair sensitivity and specificity (23, 24). Continuous and accelerated pulmonary fellow with nonphasic flow pattern is the indication of PVO. The obstruction may be caused by extrinsic pressures or intrinsic narrowing of the vein, or both (25). Previous study showed the rate of postoperative PVO is higher than 10% (8, 26). In our study, the incidence of postoperative PVO is 2.5% which is low. Though previous studies showed mixed and infracardiac TAPVC are strongly associated with postoperative PVO (8, 27). Our study showed no association between the types of TAPVC and risk of higher postoperative pulmonary vein flow velocity. We believe the primary sutureless technique may eliminate the differences between types while decrease the postoperative PVO rate, which makes it applicable in any subtypes of TAPVC.

Previous studies also showed preoperative PVO as a significant predictor for postoperative PVO (8, 28). Our study showed no consistent result. There is no significant association between preoperative PVO and higher postoperative pulmonary vein flow velocity in our study. We believe that primary sutureless technique may also eliminate the difference here for it can fully relieve the preoperative PVO. Following the satisfactory outcomes in preventing postoperative PVO in all subtypes in our study, we advocate the indications for primary sutureless repair may expand further to all the TAPVC patients.

In earlier reports, lower body weight, younger age at surgery and emergency surgery were associated with postoperative PVO (8). But in our study, these factors were no risk factors for higher postoperative pulmonary vein flow velocity. Supporting the feasibility of early surgical correction of TAPVC (29). Corrective surgery for patients with TAPVC should be performed as soon as possible (30).

We found that preoperative pneumonia (*p* = 0.022), prolonged CPB time (*p* = 0.009) and gender(male) (*p* = 0.041) were risk factors for higher postoperative pulmonary vein flow velocity. Of note, in previous study of pneumonia patients, compared with those without preoperative pneumonia, children with preoperative pneumonia had a higher risk of mortality and postoperative complications, longer hospital stay (31). It is believed that preoperative pneumonia and longer CPB time may indicate those patients who are in poor preoperative condition and poorer pulmonary condition. Therefore, they can be more difficult to operate on and recover.

The surgical mortality in early series is from 10% to 80% (3, 7, 32), but most recent reports presented a surgical mortality that is <10% (3, 4, 18). Our study, with primary sutureless technique, mortality was 3.8% which is low. 2 patients presented early deaths, according to our death case discussion record. One due to suffocation led by improper feeding by the patient's mother. One due to acute pulmonary edema which led by pulmonary hypertension. 1 late death occurred after the patient's second surgery because of the comprehensive effect of multiple conditions. Due to the small sample size of mortality cases in out study, we did not apply Cox regression or other models to analyze the risk factor for mortality. Thus, more cases and longer surveillance are required for further study.

## Limitations

The study is subject to the usual limitations of a retrospective, nonrandomized study design. Due to the small sample size of mortality cases. We did not make any statistical analysis for mortality. There are more variables we wanted to include in our study, for example, CVP, Blood Gas Analysis results. But ended up impossible for these variables cannot be traced in multiple cases. We excluded patients with single ventricle or heterotaxy. Further study of primary sutureless technique to clarify and compare the clinical course of TAPVC with single-ventricle and heterotaxy physiology is necessary. Extracardiac anomalies were not taken into the study. Further study on the comprehensive influence of extracardiac anomalies on post operative outcomes is necessary.

## Conclusions

Prolonged CPB time and preoperative pneumonia are the major risk factors of faster postoperative pulmonary vein flow velocity. The primary sutureless technique may eliminate the differences between types while decrease the postoperative PVO rate, which makes it applicable in any subtypes of TAPVC. Following the satisfactory outcomes in preventing postoperative PVO in all subtypes in our study, we advocate the indications for primary sutureless repair may expand further to all the TAPVC patients.

## Data Availability

The original contributions presented in the study are included in the article/Supplementary Material, further inquiries can be directed to the corresponding author/s.
